# PRDM14 extinction enables the initiation of trophoblast stem cell formation

**DOI:** 10.1007/s00018-024-05237-9

**Published:** 2024-05-06

**Authors:** Chunfang Xu, Weijie Zhao, Lijin Peng, Tingxuan Yin, Jiani Guo, Yue Li, Lu Liu, Jinying Yang, Congjian Xu, Meirong Du

**Affiliations:** 1 Laboratory for Reproductive Immunology, Shanghai Key Laboratory of Female Reproductive Endocrine Related Diseases, Hospital of Obstetrics and Gynecology, Fudan University Shanghai Medical College, Shanghai, 200032 China; 2 Longgang District Maternity & Child Healthcare Hospital of Shenzhen City, Longgang Maternity Child Institute of Shantou University Medical College, Shenzhen, 518172 China; 3 Department of Obstetrics and Gynecology, Shanghai Fourth People’s Hospital, School of Medicine, Tongji University Shanghai, Shanghai, 200434 China; 4 State Key Laboratory of Quality Research in Chinese Medicine and School of Pharmacy, Macau University of Science and Technology, Macau, 519020 SAR China

**Keywords:** PRDM14, Wnt, Human trophoblast stem cells, H3K27me3

## Abstract

Trophoblast stem cells (TSCs) can be chemically converted from embryonic stem cells (ESCs) in vitro. Although several transcription factors (TFs) have been recognized as essential for TSC formation, it remains unclear how differentiation cues link elimination of stemness with the establishment of TSC identity. Here, we show that PRDM14, a critical pluripotent circuitry component, is reduced during the formation of TSCs. The reduction is further shown to be due to the activation of Wnt/β-catenin signaling. The extinction of PRDM14 results in the erasure of H3K27me3 marks and chromatin opening in the gene loci of TSC TFs, including GATA3 and TFAP2C, which enables their expression and thus the initiation of the TSC formation process. Accordingly, PRDM14 reduction is proposed here as a critical event that couples elimination of stemness with the initiation of TSC formation. The present study provides novel insights into how induction signals initiate TSC formation.

## Introduction

Numerous pregnancy-associated diseases have been attributed to functional defects in the placenta, such as preeclampsia (PE), fetal growth restriction (FGR), and recurrent spontaneous abortion (RSA) [1, 2]. To gain a comprehensive understanding of how defects arise, it is a prerequisite to thoroughly investigate the differentiation process of the placenta. Following the formation of blastocysts, cells within the inner cell mass (ICM) give rise to the embryo proper, whereas the trophectoderm (TE) further differentiates to form the placenta [3]. By using the mouse model, several critical transcription factors (TFs) for trophoblast cell formation have been identified. For example, the Hippo signaling pathway is activated in the TE; as a result, the downstream transcription coactivator YAP1 translocates into the nucleus, where it functions together with TEAD4 to initiate CDX2 expression and hence the trophoblast cell differentiation program [4, 5]. Other important TFs include GATA3, TFAP2C, and HAND1 [6]. In recent years, human trophoblast stem cells (TSCs) have been obtained from primary first-trimester placenta or embryonic stem cells (ESCs) through the utilization of a cocktail of compounds and cytokines [7–9]. This major breakthrough not only provides a valuable resource to investigate the mechanisms of trophoblast cell differentiation, but also reveals important signaling needed for the differentiation process.

Despite the aforementioned progress, there have been few reports addressing the question of how stemness is eliminated by trophoblast differentiation cues. The withdrawal of stemness is crucial for the expression of lineage-associated TFs, as shown by the fact that artificial overexpression of pluripotent OSKM factors (OCT4, SOX2, KLF4, and c-Myc) can cause erasure of cell identity [10]. Trophoblast cells are the result of the first lineage allocation made by ESCs, which requires the cooperation of stemness extinction and differentiation initiation. In ESCs, the pluripotent circuitry is established, at least in part, by OCT4, SOX2, NANOG, and PRDM14 [11, 12]. Among these, PRDM14 is relatively less studied than the other three, as is its regulation during trophoblast differentiation. Therefore, we selected PRDM14 for investigation in the present study.

PRDM14 belongs to a 16-member family, which is characterized by a PR domain and multiple zinc fingers, except for PRDM11. The PR domain is a divergent form of the SET domain, which can be observed in the catalytic sites of a number of histone methyltransferases [13]. This phenomenon suggests the ability of the PRDM family to mediate methylation, which has been proven for some members (PRDM2, PRDM7, PRDM8, PRDM9, and PRDM16) but not for others (PRDM14) [14]. In ESCs, PRDM14 functions collaboratively with OCT4 and SOX2 to maintain the pluripotent state or with the PRC2 complex to repress its targets, which might comprise developmental genes [15]. The reduction in PRDM14 is largely associated with cell differentiation, with the exception of the formation of primordial germ cells (PGCs), wherein PRDM14 plays an indispensable role [16]. The expression and regulation of PRDM14 in TSC differentiation remain to be investigated.

We show here that during the induction of TSCs, Wnt signaling elicits a reduction in PRDM14, which promotes the erasure of H3K27me3 in the gene loci of TFs critical for TSC differentiation, including GATA3, TFAP2C, and HAND1, thus enabling their expression. The present study provides novel insights into how the TSC formation program is initiated by the induction signal.

## Materials and methods

### Antibodies, compounds, and cytokines

PRDM14 (#83527, Cell Signaling Technology), ACTB (AC026, ABclonal), β-catenin (ab32572, Abcam), GATA3 (ab199428, Abcam), GATA3 (ab282110, Abcam), KRT7 (ab68459, Abcam), TFAP2C (ab218107, Abcam), HAND1 (D263110, Sangon Biotech), total H3 (AF0009, Beyotime), H3K27me3 (#9733, CST), SUZ12 (#3737, CST), TACSTD2-PE (363803, BioLegend), SIGLEC6-APC (FAB2859A, R&D), goat anti-mouse IgG (H + L) (115-005-003, Jackson ImmunoResearch), goat anti-rabbit IgG (H + L) (111-005-003, Jackson ImmunoResearch), goat anti-mouse IgG H&L (Alexa Fluor® 488) (ab150113, Abcam), goat anti-rabbit IgG H&L (Alexa Fluor® 647) (ab150079, Abcam), GSK126 (T2079, TargetMol), LGK974 (T2618, TargetMol), A83-01 (HY-10432A, MCE), CHIR99021 (T2310L, TargetMol), EGF(AF-100-15, Peprotech), Y27632 (T1725, TargetMol), BMP4 (120-05, Peprotech), TSA (T6270, TargetMol), and Vitamin C (Vc) (HY-B0166, MCE) were obtained.

### Cell culture

The human ESC line H9 (WiCell) was routinely cultured in commercial E8 medium (Cellapy, China) [17] (DMEM/F12 buffered by NaHCO_3_, insulin, transferrin, sodium selenium, Vc, TGF-β1, and FGF-basic) on plates coated with the extracellular matrix Matrigel (354277, Corning). In some experiments, TGF-β1 and FGF-basic were removed from the E8 medium, resulting in the E6 medium. For passaging, cells were dissociated by 0.5 mM EDTA (GENOM, China) at 37 °C for approximately 5 min. Mediums were refreshed daily. Cells were maintained in an incubator at 37 °C and 5% CO_2_.

For TSC induction, ESCs were passaged and seeded into six-well plates precoated with Matrigel at a density of 3 × 10^5^ cells/well. Overnight, the medium was removed and the cells were washed twice with PBS. Then, the induction medium was added to the wells. After 6 days, the cells were harvested for analyses. The induction duration may be shorter than 6 days as indicated in the figures. The components of the induction medium were as follows [9]: DMEM/F12 (11320033, Gibco), 0.3% BSA (A9205, Sigma), ITS-X (51500056, Gibco), 0.2% FBS (10099141C, Gibco), 10 μM Vc, 0.5 μM A83-01, 2 μM CHIR99021, 50 ng/ml EGF, 5 μM Y27632, and 10 ng/ml BMP4.

### Generation of cell lines stably expressing PRDM14

The PRDM14 coding sequence was inserted into a piggyBAC vector, which is driven by the EF1A promoter and contains an EGFP-T2A-puroR selection cassette. This PRDM14 expression vector and the piggyBAC transposase vector were co-transfected into H9 cells using LTX plus reagent (#15338100, Invitrogen). Three days later, the cells were subjected to puromycin (2 μg/ml) selection for two days. After additional 5–7 days of culture, the resistant colonies were picked up for further propagation and experiments. Mediums were routinely refreshed daily, and cells were passaged every 4–6 days using 0.5 mM EDTA.

### siRNA Transfection

The siRNA sequences targeting the genes of interest and their corresponding scrambled controls were designed and synthesized by Shanghai Generay Biotech Co., Ltd. The sequences of the PRDM14 and β-catenin siRNAs are listed as follows.

PRDM14

Sense: GGAAGGUAUUUACCUACAATT.

Anti-sense: UUGUAGGUAAAUACCUUCCTT.

β-catenin

Sense: GAAUACAAAUGAUGUAGAATT.

Anti-sense: UUCUACAUCAUUUGUAUUCTT.

Cells were seeded into six-well plates precoated with Matrigel at a density of 3 × 10^5^ cells/well. Six hours later, siRNAs were transfected into cells using RNAiMAX (#13778150, Invitrogen). Overnight, the medium was removed, and the cells were washed once with PBS. Afterward, the induction medium or E8 medium, which was dependent on the experimental design, was added to the wells. Three days later, the cells were harvested for analyses. Mediums were refreshed daily.

### CUT&Tag-seq library preparation and data processing

The protocol was modified from the one developed by Henikoff. Briefly, cells were dissociated by Accutase (A1110501, Gibco) at 37 ℃ for approximately 5 min. Single-cell suspensions were incubated with activated ConA beads (Biolinkedin, China) at room temperature (RT) for 10 min, followed by incubation with primary antibody (2 h, RT), secondary antibody (unconjugated) (Jackson ImmunoResearch) (1 h, RT) and pAG-Tn5 preloaded with mosaic end adapters (Novoprotein, China) (1 h, RT). Afterward, these cell-attached beads were treated with MgCl_2_ to activate tagmentation. Then DNA fragments were purified by DNA extraction beads (Beyotime, China) and were subsequently amplified by a high-fidelity DNA polymerase mix (Yeason, China) to obtain the final library. Libraries were sequenced on a NovaSeq 6000 system. Raw reads were filtered and trimmed by trim-galore (v0.6.10). The clean reads were aligned to the human genome (hg38) using Bowtie2 (v2.2.5) [18]. PCR duplicates were removed by sambamba (v0.6.6). Peak calling was performed by macs2 (v2.2.6). Differential binding peaks were obtained by the R package Diffbind (v3.10.1) [19]. The intersect function of bedtools (v2.30.0) was used to analyze the peak files for recognizing co-binding events [20]. Annotations for peaks were performed by the R package ChIPseeker (v1.32.1). Genome browser views of peaks were generated by IGV software (v2.16.0).

### ATAC-seq library preparation and data processing

Cells were dissociated by Accutase (A1110501, Gibco) at 37 ℃ for approximately 5 min. Then they were lysed in a hypotonic buffer. The nuclei were then collected and subjected to tagmentation by Tn5 preloaded with mosaic end adapters (Novoprotein, China) at 37 °C for 30 min. DNA fragments were purified and amplified with a high-fidelity DNA polymerase mix (Yeason, China) to obtain the final library. Libraries were purified and sequenced on a NovaSeq 6000 system. Raw reads were filtered and trimmed by trim-galore (v0.6.10) [21]. Human genome (hg38) mapping was performed by Bowtie2 (v2.5.1). After removal of PCR duplicates by sambamba (v0.6.6), peaks were called by macs2 (v2.2.6) [22]. Differentially accessible peaks were generated by the R package Diffbind (v3.10.1). To find those peaks co-bound by the indicated TFs, we used the intersect function of bedtools. Heatmaps of the differential peaks were generated by deepTools (v3.5.1). When the replicates were not shown individually, their bam files were pooled together to generate the bw files for heatmap presentation. Annotations for peaks were performed by the R package ChIPseeker (v1.32.1) to obtain their profiles of genomic distribution and the list of the nearest genes. Genome browser views of peaks were generated by IGV software (v2.16.0).

### RT-qPCR

Total RNA was extracted by RNAiso reagent (TaKaRa). Then cDNA was synthesized using a reverse transcription kit (Yeason, China), according to the manufacturer’s guidelines. A SYBR Green-based qPCR kit (Yeason, China) was used to perform real-time quantitative PCR on an Applied Biosystem Q6. The relative mRNA expression levels of specific genes normalized to that of β-actin (ACTB) were analyzed using the 2^(−ΔΔCT)^ method. Data are presented as the mean ± SEM of three replicates. The primer sequences are shown in Table 1.

**Table 1:** List of Primers uesd for quantitative real-time PCR

Gene	Forward sequence	Reverse sequence
PRDM14	GTCCCAAACCCTCCAACCAA	TCACCAAACACCGTCTGCAT
TFAP2C	AGTCATTCGCAAAGGTCCCA	ACCGGCCTCCATTTTTCGAT
TFAP2A	CGAATCGGTGGTTCAAGTTCG	GTCGTGACGGTCCATGGCT
GATA3	GCGCCGTCTTGATACTTTCAG	TCCTCGGGTCACCTGGGTAG
GATA2	TCGTCCGAACCATCCCAAC	GCAACGGCCACACGCA
ACTB	TCTGGTGATGCTGCCATTGT	AAAGCGACCCAAAGGTGGAT
HAND1	GGGGATGGCAGGATGAACAA	AGGAAGATGAAAGGCTGCCC
SIGLEC6	TCACAACCCTGGTTTTCCTC	CTGTCTGGAACTGGTGCTGA
TACSTD2	CGGCAGAACACGTCTCAGAA	GCCCTGGAATAGAGACTCGC
ENPEP	CTTGGCAAGGTTACTCCGTT	GCCAGAGTTCTGCATCCCAT
DAB2	GTCGGTCTCAGGGACAACAC	TGTCACATCACGGGCAATGA
VGLL1	ATTGACCCCCTCGAGTCAGA	ATGGAGACGAGTAACGCCAC
EGFR	TGGTCAAGTGCTGGATGATAGA	CGGTGGAATTGTTGCTGGTT
KRT7	GACATCGAGATCGCCACCTA	ATTCACGGCTCCCACTCCAT
KRT19	AGCAGGTCCGAGGTTACTGA	CCAAGGCAGCTTTCATGCTCA
OCT4	GCTCGAGAAGGATGTGGTCC	CGTTGTGCATAGTCGCTGCT
NANOG	AGATGCCTCACACGGAGACT	TGCAGAAGTGGGTTGTTTGC◻
SOX2	AACCAGCGCATGGACAGTTA	GACTTGACCACCGAACCCAT

### Bulk RNA-seq library preparation and data processing

Total RNA was isolated using RNAiso (TaKaRa). Libraries were prepared using a TruSeq® Stranded Total RNA Library Preparation kit (Illumina). Following purification, mRNA was enriched with oligo (dT) beads and fragmented. Afterward, double-strand cDNA was synthesized and index adapters were incorporated. The products were then purified and enriched by PCR amplification to create the final cDNA library. Sequencing was performed on a NovaSeq 6000 system. Reads were filtered and mapped to the reference genome using HISAT2 (v2.0.4). Sequencing read counts were calculated using Stringtie (v.1.3.0). The R package DESeq2 was used to analyze intergroup differences in gene expression. Differentially expressed genes (DEGs) were defined as transcripts with an FDR less than 0.05. GO enrichment analysis was performed with the R package clusterProfiler.

### Western blotting

Cells were lysed in RIPA lysis buffer (50 mM Tris (pH 7.4), 150 mM NaCl, 1% (v/v) Triton X-100, 1% (w/v) sodium deoxycholate, and 0.1% (w/v) SDS) with a protein inhibitor cocktail (HY-K0010, MCE). Protein concentrations were determined by the BCA method. Equal amounts of total proteins (10 μg) were resolved by SDS-PAGE and transferred onto PVDF membranes. Then the membranes were blocked with 5% (w/v) skim milk in TBST at RT for 1 h, followed by incubation with primary antibodies prepared in TBST at 4 °C overnight. After incubation with HRP-conjugated secondary antibodies (Biosharp, China) at RT for 1 h, images were acquired with a GE Healthcare Amersham Imager 600 system. Experiments were always performed in triplicate.

### Immunocytochemistry (ICC)

Cells were seeded into confocal microscope compatible dishes (Cellvis), which were precoated with Matrigel. After preparation for scanning, cells were fixed with 4% paraformaldehyde at RT for 10 min, permeabilized with 0.1% Triton X-100 (Invitrogen) at RT for 20 min, and then blocked with 3% BSA at RT for 1 h. Cells were incubated with primary antibodies at 4 °C overnight and secondary antibodies at RT for 1 h. Afterward, cells were incubated with DAPI solution (Abcam) at RT for 5 min. Images were obtained by confocal microscope scanning microscopy(TCS SP5 II, Leica Microsytems).

### Flow cytometry

Cells were dissociated by Accutase (A1110501, Gibco) at 37 ℃ for approximately 5 min. Single-cell suspensions were treated with Human TruStain FcX™ (BioLegend) for 15 min to block non-specific Fc receptor binding. The Zombie Aqua™ Fixable Viability Kit (BioLegend) was used to discriminate between live and dead cell populations. Antibody staining was conducted in PBS for 30 min. After PBS washes, the cells were analyzed by a flow cytometer (Cytoflex, Beckman). FlowJo (v10.8.1) software was used for further analyses.

### Statistical analysis

Data analyses were performed using GraphPad Prism (v8.0) (GraphPad Software, San Diego, CA, USA). Statistical significance was determined by a two-tailed t-test for comparison of two groups. A P-value < 0.05 was considered statistically significant in all tests.

## Results

### PRDM14 expression is reduced during TSC induction

**Fig. 1:**
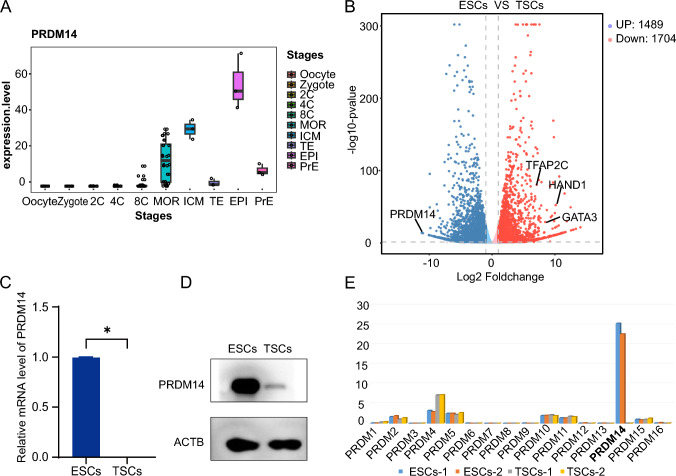
PRDM14 expression is reduced during TSC induction. **A** PRDM14 expression in fragments per kilobase million (FPKM) for the oocyte, zygote, 2 cell (2C), 4 cell (4C), 8 cell (8C), morula, inner cell mass (ICM), trophectoderm (TE), epiblast (EPI) and primitive endoderm (PrE) stages. **B** Volcano plot of differentially expressed genes (DEGs) in ESCs and TSCs (FDR < 0.05). **C** Relative mRNA expression of PRDM14 in ESCs and TSCs, normalized by that of ACTB; data are presented as the mean ± SEM of three replicates. *p < 0.05. **D** Representative Western blot for PRDM14 expression in ESCs and TSCs. **E** Histogram showing the gene expression profile of PRDM family members in ESCs and TSCs. For TSCs, ESCs were induced for 6 days.

To investigate the evolution of PRDM14 expression in the sequential pre-implantation stages of human embryos (oocyte, zygote, 2C, 4C, 8C, morula, ICM, TE, EPI and PrE), we searched several public single-cell databases for analyses [23–25]. PRDM14 expression starts from the 8C stage and can be readily detected in the morula. As soon as the blastocyst forms, its expression is almost limited to the ICM compared to the TE (Fig. 1A). Consistently, when ESCs were induced to differentiate toward TSCs, the up-regulation of TSC markers (GATA3, TFAP2C and HAND1) was accompanied by a drastic reduction in PRDM14 expression, as shown by RNA-seq analysis (Fig. 1B). This reduction was further confirmed at both the mRNA and protein levels (Fig. 1C, D). Notably, apart from PRDM14, most of the PR family members were expressed at a very low level in ESCs (Fig. 1E). According to the above information, PRDM14 expression is reduced during TSC induction.

### Wnt/β-catenin signaling causes a reduction in PRDM14 during TSC induction

**Fig. 2:**
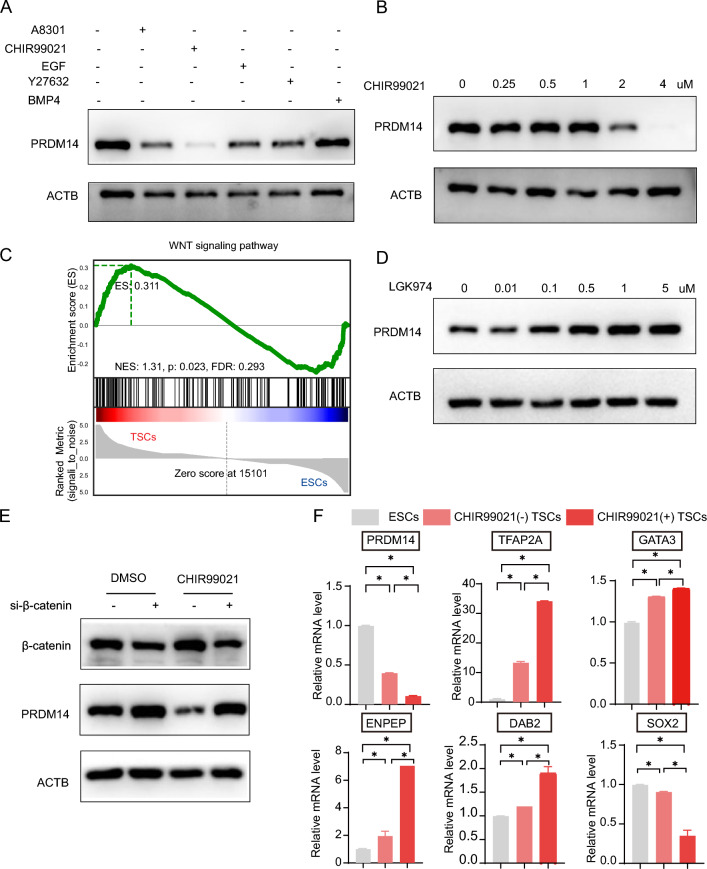
Wnt/β-catenin signaling causes a reduction in PRDM14 during TSC induction. **A** Representative Western blot for PRDM14 expression in ESCs cultured in E6 medium with the addition of A83-01, CHIR99021, EGF, Y27632 or BMP4. Cells were cultured for 48 h in these mediums. **B** Representative Western blot for PRDM14 expression in ESCs cultured in E8 medium. Cells were treated with different doses of CHIR99021 for 48 h. **C** Gene set enrichment analysis (GSEA) of Wnt signaling pathway-related genes in TSCs compared to ESCs. **D** Representative Western blot for PRDM14 expression in ESCs cultured in E8 medium. Cells were treated with different doses of LGK974 (a porcupine inhibitor for interfering with Wnt signaling) for 48 h. **E** Representative Western blot for PRDM14 and β-catenin expression in ESCs cultured in E8 medium. Cells were transfected with scrambled control siRNA or β-catenin siRNA. Overnight, the mediums were refreshed by the ones with CHIR99021 or with DMSO as control, as indicated. Another forty-eight hours later, the cells were harvested for analyses. **F** Relative mRNA expression of TSC markers and pluripotency markers. ESCs were cultured in induction medium with or without CHIR99021 for 3 days; data are presented as the mean ± SEM of three replicates, *p < 0.05.

Before the advent of the TSC induction medium developed by Okae and modified by others, it was difficult to determine how the stemness was inhibited, thus enabling the initiation of the TSC formation program. Currently, we have a list of substances that were shown to be minimally required for TSC induction, and their unique roles have begun to be revealed. By analyzing these listed compounds and cytokines separately, we found that CHIR99021 was the most efficient at reducing PRDM14 expression (Fig. 2A), and the effect was also shown to be dose-dependent (Fig. 2B). CHIR99021 is commonly used to mimic the action of Wnt signaling, and we indeed observed a significant enrichment of the Wnt signaling pathway in TSCs compared to ESCs (Fig. 2C). Notably, however, the reduction in PRDM14 cannot be simply attributed to Wnt signaling at present, as the observed effects were complicated by GSK3α, which can be equally inhibited by CHIR99021, and by those parts of the GSK3β pool in a cell that function independently of Wnt signaling. To address these concerns, we used LGK974, a porcupine inhibitor that can interfere with the palmitoylation of Wnt proteins and thus prevent the production of active Wnt ligands. It was observed that LGK974 could elevate PRDM14 expression in a dose-dependent manner (Fig. 2D). Furthermore, we showed that knocking down β-catenin in ESCs could increase PRDM14 expression and could also preserve its expression in the presence of CHIR99021 (Fig. 2E). Given that CHIR99021 was highly efficient in reducing PRDM14 expression, we reasoned that TSC induction might be accelerated as a result of a quick exit of pluripotency. Indeed, we found that compared to its absence, CHIR99021 could promote the expression of several TSC markers (GATA3, TFAP2A, DAB2, and ENPEP) and reduce the expression of SOX2, a pluripotent factor (Fig. 2F). The above results together indicated that Wnt/β-catenin signaling causes a reduction in PRDM14 during TSC induction.

### The PRDM14 reduction promotes TSC induction

**Fig. 3:**
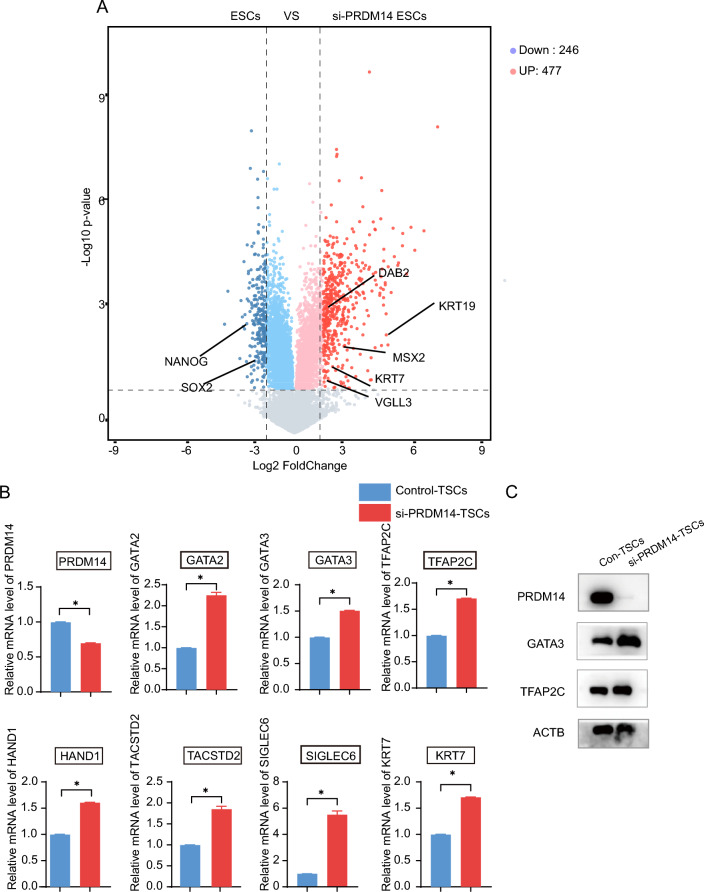
PRDM14 knockdown promotes TSC formation. **A** Volcano plot of DEGs in ESCs transfected with scrambled control siRNA or PRDM14 siRNA (FDR < 0.05). **B-C** Relative mRNA expression and representative Western blot for the indicated genes. ESCs were transfected with scrambled control siRNA or PRDM14 siRNA. Overnight, the E8 mediums were replaced with induction mediums. Three days later, the cells were harvested for analyses. Data are presented as the mean ± SEM of three replicates. *p < 0.05.

**Fig. 4:**
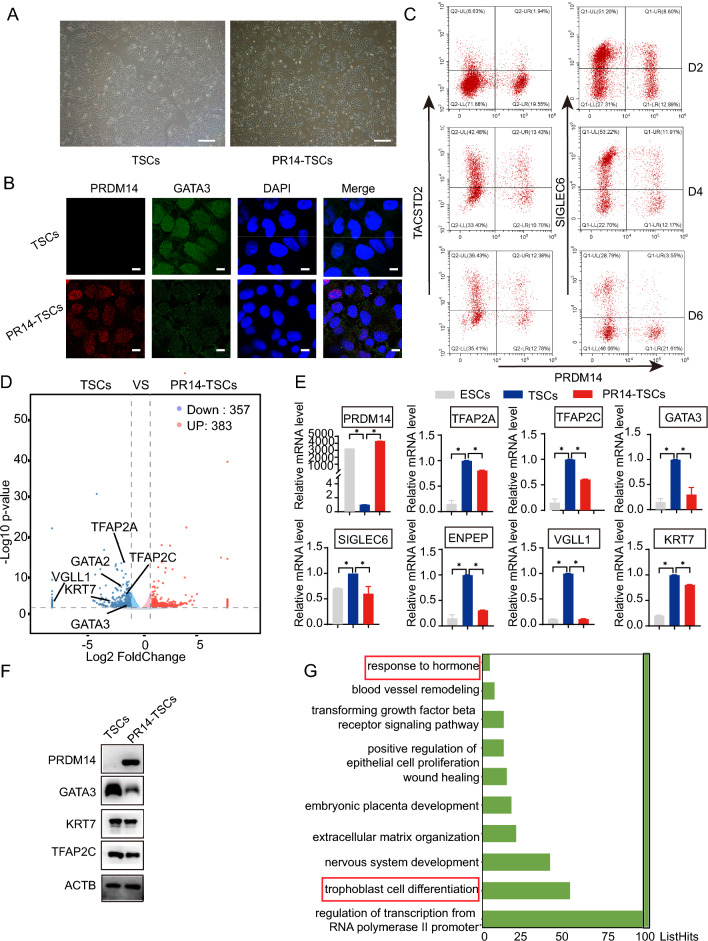
ESCs stably expressing PRDM14 fail to differentiate toward TSCs. **A** Phase-contrast images of TSCs on Day 6 induced from ESCs without transfection or ESCs stably expressing PRDM14 (PR14-ESCs). Scale bars, 200 μm. **B** Immunofluorescence images showing GATA3 expression in TSCs on Day 6, induced from ESCs or PR14-ESCs. Scale bars, 50 μm. **C** Flow cytometry analysis of TACSTD2 and SIGLEC6 expression in TSCs on Days 2, 4, and 6 induced from ESCs or PR14-ESCs. **D** Volcano plot of DEGs based on RNA-seq analyses between TSCs induced from ESCs and those from PR14-ESCs (FDR < 0.05). **E** Relative mRNA expression of the indicated genes in ESCs and TSCs induced from ESCs or PR14-ESCs, normalized by that of ACTB; data are presented as the mean ± SEM of three replicates, *p < 0.05. **F** Representative Western blot for the indicated proteins in TSCs induced from ESCs or PR14-ESCs. **G** The top 10 terms identified by Gene Ontology (GO) enrichment analysis in the downregulated gene set in TSCs induced from PR14-ESCs.

The question of whether PRDM14 reduction is a necessary event for TSC induction remains to be answered. To this end, we first explored a set of microarray data published in an early study in which the effects of PRDM14 knockdown on ESCs were analyzed [26]. Several trophoblast-associated genes, including KRT7, KRT19 and DAB2, were induced when PRDM14 was silenced, indicating partial initiation of the TSC formation program (Fig. 3A). This observation suggested that reduced PRDM14 might be beneficial for TSC induction. Indeed, when PRDM14 was knocked down by siRNA, TSC markers displayed higher expression during TSC induction (Fig. 3B, C). To further ascertain the importance of PRDM14 reduction in TSC formation, we established an ESC line stably expressing PRDM14 (PR14-ESCs) by using the piggyBAC transposon system. When cultured in E8 medium, this cell line proliferated normally and showed no discernible morphological alterations compared to the parental cell line. When induced for TSC differentiation, ESCs underwent extensive changes in their shapes and presented the canonical flattened epithelial-like morphology, whereas these changes were marginally observed in PR14-TSCs induced from PR14-ESCs (Fig. 4A). As shown in Fig. 4B, GATA3, a critical TF for TSC formation, showed robust expression after 6 days of induction, whereas it was almost undetectable in the presence of PRDM14. For further evaluation of the effects of PRDM14 expression on TSC formation, PR14-ESCs, which also carried an EGFP expression cassette, and parental ESCs were mixed together and induced concurrently toward TSCs. In an analysis by flow cytometry, SIGLEC6 and TACSTD2, two transmembrane proteins highly expressed in TSCs, were shown to be strongly induced in parental ESCs; in contrast, their expression was severely diminished in the presence of PRDM14 (Fig. 4C). We also performed RNA-seq analysis, and consistently, the expression of the aforementioned genes and several additional markers for TSCs was found to be repressed in the presence of PRDM14 (Fig. 4D), these results were also confirmed by qPCR and Western blot analysis (Fig. 4E, F). Moreover, Gene Ontology (GO) enrichment of DEGs showed terms related to hormone responsiveness and placental development (Fig. 4G). Collectively, these observations indicated that PRDM14 reduction favors TSC induction.

### The PRDM14 reduction enables chromatin accessibility remodeling during TSC induction

**Fig. 5:**
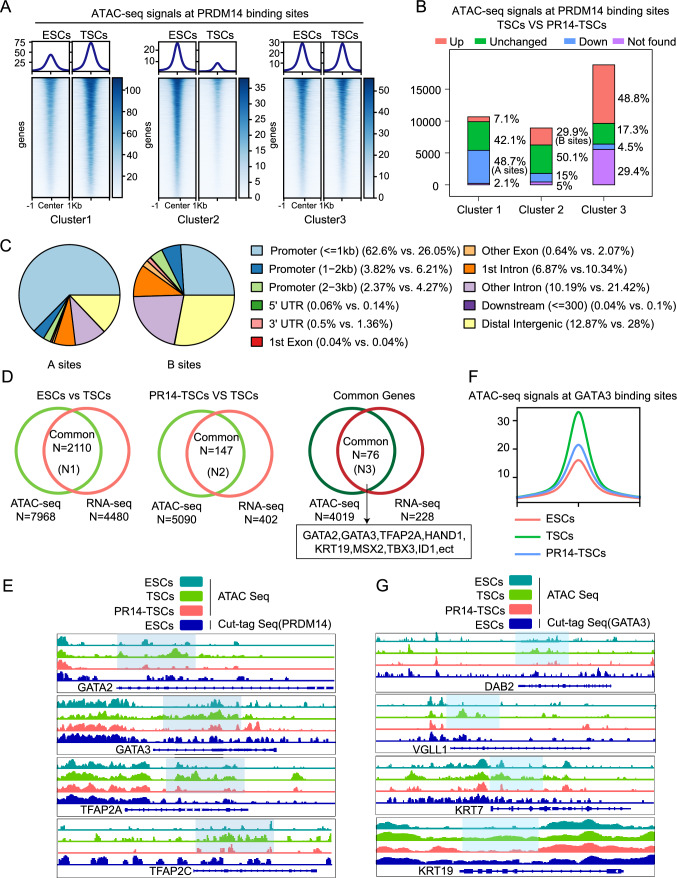
PRDM14 reduction enables chromatin accessibility remodeling during TSC induction. **A** Density plot and heatmap showing chromatin accessible signals around PRDM14 binding sites that were identified in ESCs. With Diffbind, the sites were classified into Cluster 1, Cluster 2, and Cluster 3, which had respectively with higher accessibility in TSCs, ESCs, or with equal accessibility in both of them. **B** Accessibility changes in the clusters described in (A) in the presence of PRDM14, obtained by comparing their signals in TSCs induced from PR14-ESCs with those from the parental ESCs. Those sites showing downward signals in Cluster 1, and upward signals in Cluster 2 were denoted as A sites and B sites respectively. **C** Genomic distribution of A sites and B sites, as described in (B). **D** Venn diagram indicating the number of overlapping genes between those next to the ATAC-seq peaks with higher signals and those showing higher mRNA expression in TSCs compared to ESCs (N1) or TSCs induced from PR14-ESCs (N2). Common genes of N1 and N2 were designated N3. **E** IGV view showing ATAC-seq signals and PRDM14 binding signals obtained by CUT&Tag-seq analyses. **F** Density plot showing ATAC-seq signals around GATA3 binding sites identified in TSCs. **G** IGV view showing ATAC-seq signals and GATA3 binding signals obtained by CUT&Tag-seq analyses.

We performed ATAC-seq analyses to gain insight into how PRDM14 reduction promoted TSC induction. The differentially accessible sites obtained by comparing ESCs and TSCs intersected with the PRDM14 binding sites identified in ESCs. The sites exhibiting higher, lower, and equal accessibilities in TSCs were classified into Cluster 1, Cluster 2, and Cluster 3 respectively (Fig. 5A). These clusters subsequently intersected with the differentially accessible sites between TSCs and TSCs induced from PR14-ESCs. As shown in Fig. 5B, in the presence of PRDM14, 48.7% of the sites with higher accessibilities in TSCs (Cluster 1) became less accessible (A sites), whereas 29.9% of the sites programmed to be closed (Cluster 2) failed to do so (B sites) (Fig. 5B). These alterations were thought to reversely reflect the outcomes of PRDM14 reduction. Genomic distribution analyses revealed that A sites contained a large number of gene promoters, whereas B sites were mainly involved distal intergenic regions and introns (Fig. 5C). These observations suggested that chromatin regions, probably including those harboring TSC genes, might be rendered more accessible, thus allowing gene expression; in addition, regulatory elements normally functioning in ESCs might be permanently closed. The presence of PRDM14 was proposed to obstruct these expected alterations.

To confirm these suggestions, we performed joint analyses, with the addition of RNA-seq data. Among those genes near to the sites that displayed higher accessibility in TSCs, 2110 of them showed upward expression (N1); 147 of the genes annotated proximal to the sites that were less accessible in the presence of PRDM14 exhibited a reduction in their expression (N2). Importantly, TFs and other marker genes of TSCs could be found in both N1 and N2 (N3) (Fig. 5D, E). We took GATA3 for further investigations, as this molecular was shown to be a pioneer factor and possess the ability to bind to and open closed chromatin regions. We expected that due to the presence of PRDM14, reduced expression of GATA3 would result in failure of chromatin opening around its binding sites as defined in TSCs (GATA3 sites). Indeed, as shown in Fig. 5F, GATA3 sites were rendered more accessible during induction, whereas this effect did not occur in the presence of PRDM14. Specifically, these observations were consistently reflected in the gene loci that harbor GATA sites (Fig. 5G). Taken together, we concluded that PRDM14 reduction enables chromatin accessibility remodeling during TSC induction.

### The PRDM14 reduction results in H3K27me3 erasure in gene loci of trophoblast TFs

**Fig. 6:**
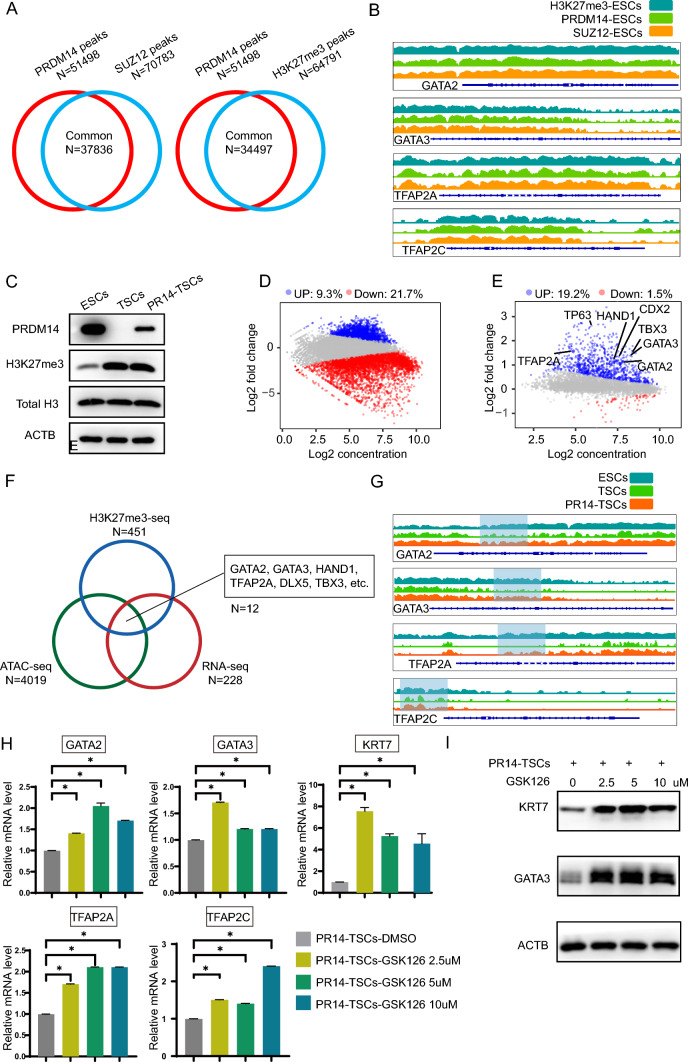
PRDM14 reduction causes H3K27me3 erasure in the gene loci of trophoblast TFs. **A** Venn diagram indicating the number of overlapping peaks between PRDM14 and SUZ12/H3K27me3 in ESCs. **B** IGV view showing that the binding signals of H3K27me3, PRDM14, and SUZ12 were concurrently detected by CUT&Tag-seq analyses around the gene loci of GATA2/3 and TFAP2A/C in ESCs. **C** Representative Western blot showing protein expression levels in ESCs, TSCs, and TSCs induced from PR14-ESCs. **D** Scatterplot showing differential H3K27me3 signals at PRDM14 binding sites identified in ESCs. By using Diffbind, the sites with higher, lower, or equal signals in TSCs compared with those in ESCs (TSC vs ESC) were designated as the blue, red, or gray respectively. FDR < 0.05. **E** Scatterplot showing differential H3K27me3 signals at the subset of PRDM14 binding sites that showed lower H3K27me3 signals in TSCs (red sites in (D)). By using Diffbind, the sites with higher, lower, or equal signals in TSCs induced from PR14-ESCs compared with those in normally induced TSCs (PR14-TSC vs TSC) were designated blue, red, or gray respectively. TFs that were nearest to the analyzed sites were designated. FDR < 0.05. **F** Venn diagram indicating the number of overlapping genes between N3 as designated in Fig. 5D and those next to the PRDM14 binding sites with lower H3K27me3 signals in TSCs than in ESCs and concurrently with higher H3K27me3 signals in TSCs induced from PR14-ESCs than in those normally induced TSCs. **G** IGV view showing H3K27me3 signals around the GATA2/3 and TFAP2A/C gene loci obtained by CUT&Tag-seq analyses. **H–I** Relative mRNA expression and representative Western blot for the indicated genes. PR14-ESCs were cultured in induction medium with various doses of GSK126 for 6 days; data are presented as the mean ± SEM of three replicates. *p < 0.05.

We have shown that PRDM14 reduction renders the chromatin more accessible in the gene loci of trophoblast TFs. This process might be achieved by the action of the first-wave TFs activated by the induction cue, resulting in nucleosome sliding and ejection and thus exposure of transacting elements. However, the observation that the presence of PRDM14 precluded these proposed alterations indicated that the reduction in PRDM14 was a prerequisite for any ensuing events. The occupancy of PRDM14 might be an obstacle for the binding of other TFs, notably pioneers. In this regard, it was more likely that additional TFs or complexes could be recruited and together formed a repressive machinery, exemplified by the PRC2 complex, which comprised several core subunits (EZH2, SUZ12, EED and RbAP) and additional accessary subunits and drove H3K27me3 deposition. Indeed, it has been reported that PRDM14 is able to recruit the PRC2 complex to repress its targets [27]. We examined the binding profile of PRDM14 in ESCs and compared them with those of SUZ12 and H3K27me3. Importantly, 73.4% of the PRDM14 peaks overlapped with those of SUZ12 and 66.9% overlapped with those of H3K27me3 (Fig. 6A). Specifically, their binding events were found to coexist in gene loci of critical TSC TFs (GATA2/3, TFAP2A/C) (Fig. 6B), which indicated that the TSC induction program was kept silenced by a repressive machinery governed by PRDM14. We further explored the alterations of H3K27me3 marks in PRDM14 binding sites during TSC induction. Although there appeared to be an overall increase in H3K27me3 levels (Fig. 6C), we clearly observed that 21.7% of PRDM14 binding sites with H3K27me3 marks exhibited a downward signal intensity (D sites); in contrast, the number was 9.3% for those sites showing an increase in binding events (U sites) (Fig. 6D). Furthermore, in the presence of PRDM14, most of the D sites with differential H3K27me3 binding signals exhibited an upward trend (Fig. 6E). Notably, this kind of site comprised those residing in the GATA2/3, TFAP2A, and HAND1 gene loci; as mentioned above, these gene loci also became less accessible in the presence of PRDM14, accompanied by reduced gene expression (Fig. 6E–G). These observations indicated that PRDM14 reduction was essentially needed for H3K27me3 mark re-configuration, thereby allowing the expression of trophoblast TFs. In addition, given that PRDM14 promoted H3K37me3 deposition to produce repressive effects, we reasoned that a PRC2 inhibitor might be able to buffer against them. Indeed, we observed that GSK126, an EZH2 inhibitor, could rescue the expression of trophoblast genes (Fig. 6H–I). Overall, we concluded that the PRDM14 reduction caused H3K27me3 erasure in the gene loci of trophoblast TFs, which enabled TSC formation.

## Discussion

While TSCs can be induced from ESCs, the mechanism by which induction signals initiate the differentiation process remains to be elucidated. Here, we show that PRDM14, a critical player in the pluripotent circuitry, was reduced by Wnt/β-catenin signaling, enabling remodeling of chromatin accessibility, H3K27me3 erasure in gene loci of GATA3/TFAP2A and other important TFs, and eventually the formation of TSCs.

The regulation of PRDM14 expression has barely been reported. By analyzing the components of the induction medium, we fortuitously uncovered that Wnt/β-catenin signaling has a regulatory role. This relationship forced us to reconsider the function of CHIR99021 in iPSC and naïve ESC induction, wherein it is commonly used [28, 29]. It is putative that Wnt/β-catenin signaling may be activated in the presence of CHIR99021 and therefore a low expression level of PRDM14. This result is seemingly contradictory, as PRDM14 has been shown to be important for pluripotent establishment and maintenance. Further investigations, therefore, are required to clarify whether PRDM14, with a pluripotent role, is replaced by other unknown factors in the medium when CHIR99021 is used. Nevertheless, a recent report that claimed a superior naïve ESC induction protocol had excluded the use of CHIR99021, and even more surprisingly, a Wnt inhibitor had been incorporated [30]. It is possible that a high expression level of PRDM14 is induced as a result of Wnt signaling inhibition, which may partly explain the reason for the superiority.

The observation of Wnt/β-catenin signaling reducing PRDM14 expression is seemingly unusual, as the activation of this pathway is well linked to transcriptional activation. On cue of activation, β-catenin translocates into the nucleus, where it binds the repressor TCFs and converts them to activators. Basically, TCFs are considered the major effector of Wnt/β-catenin signaling. Whether β-catenin can function in the opposite way, that is, bind and convert some unknown activators to repressors has not been established and need further investigations. Given that Wnt/β-catenin signaling can cause the expression of some repressors, which in turn can exert repressive effects on other genes [31], it is proposed that Wnt/β-catenin signaling might reduce PRDM14 expression in an indirect manner.

The cocktail of BAP (BMP4/A83-01/PD173074) had been used for a while to obtain TSCs before the advent of Okae's protocol, of which the efficiency was later shown to be enhanced by the addition of BMP4 [32]. As there is no CHIR99021 in the BAP medium, a self-evident question is whether it is the activation of Wnt signaling that is necessary for TSC induction or the reduction of PRDM14, which can also be achieved by A83-01, albeit with lower efficiency than CHIR99021 as demonstrated in this study. Given that overexpression of PRDM14 was demonstrated to inhibit the expression of several TSC markers, it appears that PRDM14 reduction is required, regardless of the cause. However, based on our experiences, further EVT induction can only be achieved in TSCs obtained by Okae's protocol, not BAP. This information suggests that the roles of Wnt signaling in TSC induction are far beyond the regulation of PRDM14 expression and need further investigation.

Among pluripotency factors, PRDM14 is unique in that it can recruit PRC2 repressive machinery to specific sites such as GATA3 as revealed here. The repression seems to be highly temporary and reversible, as the differentiation cue for human TSC can cause PRDM14 reduction and erasure of H3K27me3 marks. It was also noticed that the binding of PRDM14 and the alterations of H3K27me3 marks largely concern the gene body in GATA3 gene loci, but not the promoter. These pieces of information give details of how PRDM14 guarantees the silence of GATA3 and thus the TSC formation program, and on the other hand, a quick response to the differentiation cue. It is proposed the transcription actually had initiated but paused where the PRDM14 and the repressive complex resided. Whether this proposed notion is the case and is applicable for other lineage determining factors is to be investigated, but in the regard of TSCs, it can be expected that a highly responsive machinery is desired, as it is extremely urgent for an embryo to initiate the differentiation program to ensure a successful implantation.

PRDM14 was shown here to induce chromatin condensation and promote repressive H3K27me3 deposition in regions where TSC TFs, such as GATA3 and TFAP2A, reside, thereby preventing their expression and TSC induction. This function indicates its potential involvement in the process of cell identity transformation [33]. The TFs governing the cell identity of one kind should be silenced, thereby enabling the establishment of another type. In this regard, it has been shown that PRDM14 can enhance the efficiency of reprogramming of human fibroblasts in conjunction with OCT4, SOX2, and KLF4, whereas knockdown of PRDM14 impedes this process [26]. PRDM14 was also shown to be highly expressed in numerous cancers [34], but its precise roles in tumor formation and metastasis are unclear. As the concept of epithelial-to-mesenchymal transition, which was proposed to explain the origin of invasion and migration of an epithelial cancer cell, also entails identity transformation [35], whether the aberrant expression of PRDM14 initiates the erasure of epithelial cell identity is worthy of exploration. Of note, GATA3, which was shown here to be repressed by PRDM14, is also a major player in the formation of ectoderm and has been shown to be functionally silenced in numerous epithelial cancer cells [36, 37]. Whether its regulation by PRDM14, which normally occurs in ESCs, is hijacked in cancer cells requires further analysis. Given that PRDM14 reduction can be achieved by the use of CHIR99021, as shown here, it will be interesting to examine whether this regulation machinery can be used for therapeutic purposes.

## Conclusions

**Fig. 7:**
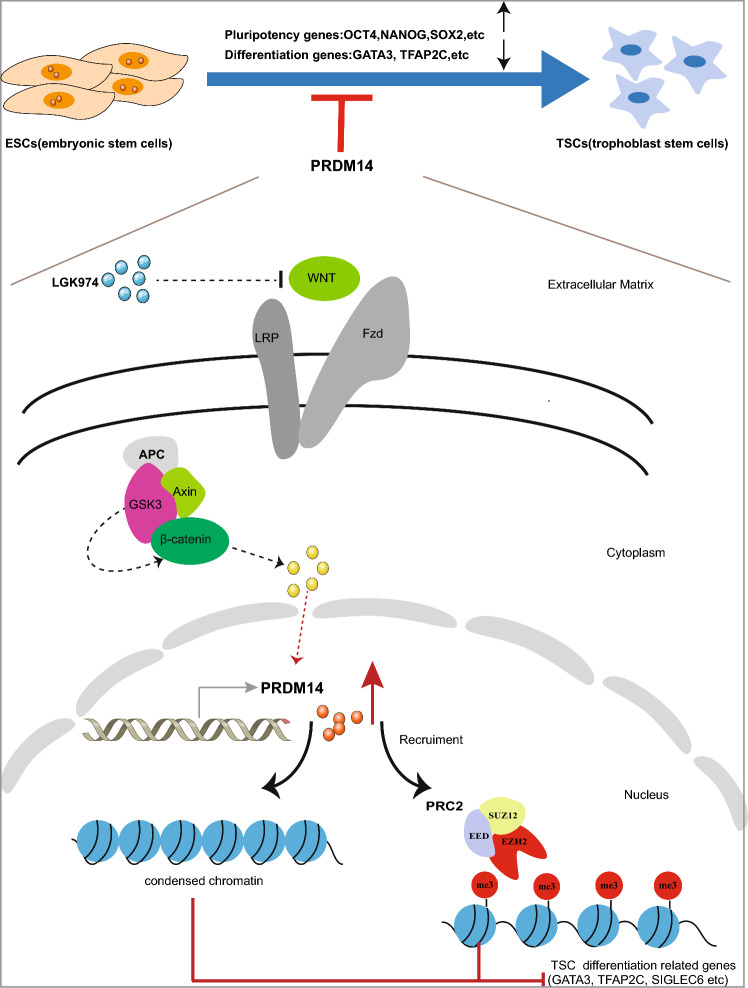
Schematic diagram showing that PRDM14 reduction is crucial for proper formation of trophoblast stem cells (TSCs). PRDM14 expression is decreased substantially during TSC formation. The Wnt signaling pathway facilitates the downregulation of PRDM14 and promotes TSC formation. Stably expressing PRDM14 or treatment with LGK974, a Wnt signaling pathway inhibitor, leads to an increase in the expression of PRDM14, which dampens the accessibility of TSCs differentiation-related genes. In addition, stably expressing PRDM14 recruits PRC2, which mediates H3K27me3 deposition and transcriptional repression of TSCs differentiation-related genes.

In summary, the present study revealed that Wnt/β-catenin signaling can elicit PRDM14 reduction, rendering it possible for chromatin accessibility re-configuration, removal of H3K27me3 marks and the expression of GATA3, TFAP2A, and other important TFs (Fig. 7). The aforementioned events should be considered as the initial stages in the process of TSC induction. Our results thus provide novel insights into how induction signals initiate trophoblast cell differentiation.

## Data Availability

The datasets used and/or analyzed during the current study are available from the corresponding author on reasoned request.

## Abbreviations

TSCs: Trophoblast stem cells
ESCs: Embryonic stem cells
TFs: Transcription factors
PE: Preeclampsia
FGR: Fetal growth restriction
RSA: Recurrent spontaneous abortion
TE: Trophectoderm
ICM: Inner cell mass
ICC: Immunocytochemistry
FPKM: Fragments per kilobase million
2C: 2 Cell
4C: 4 Cell
8C: 8 Cell
EPI: Epiblast
PrE: Primitive endoderm
DEGs: Differentially expressed genes
GSEA: Gene set enrichment analysis
PRC2: Polycomb repressive complex2
H3K27me3: Trimethylation of H3 on lysine 27
